# Collection and trade of wild-harvested orchids in Nepal

**DOI:** 10.1186/1746-4269-9-64

**Published:** 2013-08-31

**Authors:** Abishkar Subedi, Bimal Kunwar, Young Choi, Yuntao Dai, Tinde van Andel, Ram P Chaudhary, Hugo J de Boer, Barbara Gravendeel

**Affiliations:** 1 Naturalis Biodiversity Center, Sylviusweg 72, P.O. Box 9517, Leiden, The Netherlands; 2 Local Initiatives for Biodiversity, Research and Development (LI-BIRD), P.O. Box 324, Pokhara, Nepal; 3 Central Department of Botany, Tribhuvan University, Kirtipur, Nepal; 4 Institute Biology Leiden, Einsteinweg 55, 2333 CC Leiden, The Netherlands; 5 Department of Organismal Biology, Uppsala University, Norbyvägen 18 D, SE-75236 Uppsala, Sweden

**Keywords:** Commercialization, DNA barcoding, Orchids, CITES, Traditional medicine

## Abstract

**Background:**

Wild orchids are illegally harvested and traded in Nepal for use in local traditional medicine, horticulture, and international trade. This study aims to: 1) identify the diversity of species of wild orchids in trade in Nepal; 2) study the chain of commercialization from collector to client and/or export; 3) map traditional knowledge and medicinal use of orchids; and 4) integrate the collected data to propose a more sustainable approach to orchid conservation in Nepal.

**Methods:**

Trade, species diversity, and traditional use of wild-harvested orchids were documented during field surveys of markets and through interviews. Trade volumes and approximate income were estimated based on surveys and current market prices. Orchid material samples were identified to species level using a combination of morphology and DNA barcoding.

**Results:**

Orchid trade is a long tradition, and illegal export to China, India and Hong Kong is rife. Estimates show that 9.4 tons of wild orchids were illegally traded from the study sites during 2008/2009. A total of 60 species of wild orchids were reported to be used in traditional medicinal practices to cure at least 38 different ailments, including energizers, aphrodisiacs and treatments of burnt skin, fractured or dislocated bones, headaches, fever and wounds. DNA barcoding successfully identified orchid material to species level that remained sterile after culturing.

**Conclusions:**

Collection of wild orchids was found to be widespread in Nepal, but illegal trade is threatening many species in the wild. Establishment of small-scale sustainable orchid breeding enterprises could be a valuable alternative for the production of medicinal orchids for local communities. Critically endangered species should be placed on CITES Appendix I to provide extra protection to those species. DNA barcoding is an effective method for species identification and monitoring of illegal cross-border trade.

## Introduction

### Medicinal orchids of Nepal

Orchids are long known for their medicinal value. It is believed that the Chinese were the first to cultivate, describe and use orchids as early as 200 BC [1-3]. In the Indian subcontinent, the Ayurvedic medicinal system uses formulations based on orchid species. *Ashtavarga*, a group of eight medicinal plants includes four orchid ingredients, *Habenaria edgeworthii* Hook.f. ex Collet, *H*. *intermedia*, *Malaxis acuminata* D. Don, and *M*. *muscifera*^a^[3-5].

Wild orchids in Nepal are popularly known by the vernacular name *Sungava* or *Sunakhari*, which refers to their shiny yellow pseudobulbs. A total of 377 species belonging to 100 genera have been reported from Nepal, including 12 endemic species [6]. Due to inaccessibility of modern health care facilities, about 80% of the population of the country still depends on a wide range of locally available medicinal plants for their basic primary healthcare [7]. By 2004, over 590 studies related to ethnobotany in Nepal had been published [8]. Most of these studies lack detailed knowledge on local therapeutic uses of Nepalese orchids or describe very few cases only. In contrast to many other plant families, a comprehensive and detailed study of medicinal orchids in Nepal is still lacking [9].

### Trade of wild orchids for medicinal and other commercial purposes in Nepal

Trade in wild harvested orchids threatens local biodiversity due to overexploitation and habitat destruction [10]. Vaidya et al. [11] reported that around five tons of tubers of *Orchis latifolia* L. were harvested every year in Nepal to prepare ‘*Salep*’ for export at an approximatele value of USD 900 per ton. The export of valuable medicinal species such as *Dactylorhiza hatagirea* and *Gastrodia elata* from Nepal to China began in the late 1990s, and continues until today despite attempts to ban trade in these endangered species [12]. Bailes [13] reported in 1985 that in eastern Nepal about 100 trucks with 8 tons capacity each, loaded with wild-collected orchids were shipped to India illegally to prepare various *Ayurvedic* products. These cases all show that wild orchids from Nepal are popular trade items. The unsustainable use of orchid resources and illegal export of commercially important species causes biodiversity erosion and revenue loss to Nepal [12].

At present, it is difficult to come up with alternative, more sustainable methods to exploit wild Nepalese orchids. Efforts are hampered by three main problems. First of all, many illegally traded orchids cannot be identified to species level as they are often collected and traded sterile. Secondly, expertise in Nepal for artificial propagation of wild orchids is still very limited. Thirdly, surprisingly few studies from Nepal have been published on the trade of wild-collected orchids despite growing concerns on overexploitation and illegal national and cross-border trade [14,15].

This study aims to: i) identify the diversity of species in illegal trade in Nepal; ii) map traditional knowledge and medicinal use of wild orchids; iii) study the chain of commercialization from collector to client and/or export; iv) evaluate the efficacy DNA barcoding for orchid identification; and v) integrate the collected data to propose a more sustainable approach to orchid conservation.

## Materials and methods

### Study areas

**Figure 1:**
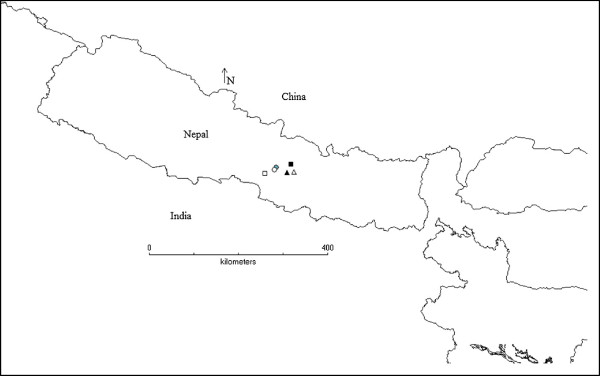
Map showing the study sites in Nepal (● Agra VDC, ▲ Dakshinkali, ∆ Godavari, ○ Gogane VDC, ■ Kathmandu, □ Manhari VDC).

Surveys were carried out in three villages in Makwanpur district of central Nepal: Agra, Gogane, and Manahari, and in two markets in the Kathmandu valley: Dakshinkali and Godavari (Figure 1). Surveys were carried out in February-March 2008 and August-November 2009.

### Data collection

Primary data were collected from interviews with local villagers involved in orchid collection, middlemen, vendors, local traders and district forest officials. We used a semi-structured questionnaire for the interviews. A detailed inventory of medicinal orchids and their uses in Nepal was prepared by a literature study. Additional information was collected through key informant interviews with local plant healers at the study sites after agreeing to a prior informed consent (PIC) on mutually agreed terms (MAT). A total of 31 people were interviewed.

### Plant identification

Wild collected flowering orchids were identified using standard literature [16-18] and cross-referenced with herbarium specimens deposited at Tribhuvan University Central Herbarium (TUCH). Sterile plants were purchased, and small cuttings were cultivated to bloom in an experimental garden in the vicinity of Pokhara for subsequent identification to species level. If no flowering could be initiated, DNA barcoding was applied. Voucher specimens of all orchid species are deposited at TUCH (Table 1).

**Table 1:** Orchids reported in traditional medicine and commercial trade in Nepal

**Scientific name voucher number**^**a**^	**Local name**	**Parts used**^**b**^	**Traditional use**	**Reference**	**Commercial trade**
*Acampe praemorsa* (Roxb.) Blatt. & McCann (syn. *Acampe papillosa* (Lindl.) Lindl.) Subedi 170	Parajivi, Rasna (Sanskrit)	Ra	Powder used in treating rheumatism and for cooling effect.	This study	Medicinal
*Aerides multiflora* Roxb. Kunwar 101	Parajivi, Thuur	Lf	Powder used in tonic preparation.	This study	Floricultural, medicinal
*Aerides odorata* Lour. Subedi 172	Parajivi	Lf	Paste of leaves used externally to treat wounds.	This study	Floricultural, medicinal
*Brachycorythis obcordata* (Lindl.) Summerh. Subedi 150	Gamdol	Tb	Powder mixed with milk and consumed as tonic.	[19]	Medicinal
*Bulbophyllum careyanum* (Hook.) Spreng. Subedi 220	Banharchul, Thuur, Parajivi	Lf, Pb	Fresh pulp of pseudobulbs externally applied to burns. Powder of leaves used with honey to induce abortions within 3 months of pregnancy and stimulate recovery from childbirth.	[10], This study	Medicinal
*Bulbophyllum leopardinum* (Wall.) Lindl. ex Wall. Subedi 221	Thuur, Parajivi	Lf, Pb	Fresh pulp or juice externally applied to burns.	This study	Medicinal
*Bulbophyllum odoratissimum* (Sm.) Lindl. ex Hook. f. Subedi 370	Thurjo	Ep	Powder used in treating tuberculosis, chronic inflammation and fractures.	[20]	Medicinal
*Calanthe sylvatica* (Thouars) Lindl. Subedi 153	Pakha phul	Fl	Juice applied to stop nosebleeds.	[11]	Floricultural, medicinal
*Calanthe plantaginea* Lindl. Kunwar 120	Ban aduwa	Rz	Dry powder consumed with milk as tonic and as aphrodisiac.	This study	Floricultural, medicinal
*Calanthe puberula* Lindl. Subedi 223	Ban aduwa	Rz	Dry powder consumed with milk as tonic and as aphrodisiac.	This study	Floricultural, medicinal
*Coelogyne corymbosa* Lindl. Subedi 375	Chadigava	Pb	Paste applied to the forehead to relieve headaches, fresh juice applied to burns as analgesic.	[7,11,21]	Floricultural, medicinal
*Coelogyne cristata* Lindl. Subedi 224	Chandigava, Bankera	Pb	Freshly collected paste or juice consumed to relieve headaches, fever and for indigestion. Pulp applied to burnt skin. Juice also applied to skin boils and wounded hooves of cattle.	[1,10,21,22], This study	Floricultural, medicinal
*Coelogyne fimbriata* Lindl. Subedi 225	Jiwanti (Sanskrit)	Pb	Powder used in tonic preparation.	This study	Floricultural, medicinal
*Coelogyne flaccida* Lindl. Subedi 301	Chadigava	Pb	Paste applied externally or consumed to relieve frontal headaches. Juice taken for indigestion.	[7,21]	Floricultural, medicinal
*Coelogyne fuscescens* Lindl. Subedi 312	Bankera	Pb	Paste applied externally or consumed to relieve headaches, fever and stomach ache. Paste applied externally on burns.	This study	Floricultural, medicinal
*Coelogyne nitida* (Wall. ex D.Don) Lindl. Subedi 226	Banlasun, Thuur	Pb	Paste consumed against headaches and fever. Paste applied externally on burns.	This study	Floricultural, medicinal
*Coelogyne prolifera* Lindl. Subedi 227	Thuur	Pb	Paste consumed against headaches and fever. Paste applied externally on burns.	[10]	Floricultural, medicinal
*Coelogyne stricta* (D.Don) Schltr. Subedi 314	Banpyaj	Pb	Paste applied externally against headaches and fever.	This study	Floricultural, medicinal
*Crepidium acuminatum* (D.Don) Szlach. (syn. *Malaxis acuminata* D. Don) Subedi 321	Gachno, Gavndamala	Ra, Pb	Powder of roots used against burning sensations, to treat fever and to stop bleeding.	This study	Medicinal
*Cymbidium aloiflolium* (L.) Sw. Subedi 228	Banharchul, Kamaru, Harjor	Ep	Dried powder used as tonic against diarrhea. Fresh paste applied externally over fractured or dislocated bones.	[10,23]	Floricultural, medicinal
*Cymbidium elegans* Lindl. Kunwar 123	Thuur	Ra, Pb	Fresh juice of pseudobulb consumed to relieve fever. Boiled root juice fed to livestock suffering from cold.	[21,22]	Floricultural, medicinal
*Cymbidium iridioides* D. Don Subedi 315	Thuur	Pb, Lf	Powder of pseudobulb consumed as tonic. Leaf juice applied externally to stimulate blood clotting in deep wounds.	[11], This study	Floricultural, medicinal
*Cypripedium himalaicum* Rolfe Kunwar 124	Khujukpa	Ep	Powder and juice consumed for urine retention, against kidney stones, heart disease, chest disorders and coughs.	[24]	Medicinal
*Dactylorhiza hatagirea* (D.Don) Soo Kunwar 103	Paanchaunle, Hatajadi	Tb	Paste consumed against fever. Powder used topically as hemostatic, or to heal fractures. Decoction consumed against intestinal pain. Tuber, eaten raw or as tonic, or mixed with honey or milk used as stimulant.	[7,23]	Medicinal
*Dendrobium amoenum* Wall. ex Lindl. Subedi 400	Thuur	Pb	Fresh paste applied topically on burnt skin and dislocated bones.	This study	Medicinal
*Dendrobium densiflorum* Lindl. Subedi 316	Sungava	Pb	Fresh pulp applied to boils and pimples.	[21,22]	Floricultural, medicinal
*Dendrobium eriiflorum* Griff. Kunwar 104	Thurjo	Pb	Paste mixed with wheat flour and applied on dislocated or fractured bones. Dried powder used as tonic.	This study	Floricultural, medicinal
*Dendrobium heterocarpum* Wall. ex Lindl. Subedi 317	Thuur	Pb	Paste mixed with wheat flour and applied on fractured or dislocated bones.	This study	Floricultural, medicinal
*Dendrobium longicornu* Lindl. Subedi 401	Kause	Ra, Pb	Juice of stems is consumed against fever. Boiled root fed to livestock suffering from coughs.	[7]	Floricultural, medicinal
*Dendrobium transparens* Wall. ex Lindl. Subedi 402	Parajivi, Thuur	Pb	Paste used on fractured or dislocated bones.	[10]	Floricultural, medicinal
*Dienia cylindrostachya* Lindl. (syn. *Malaxis cylindrostachya* (Lindl.) Kuntze) Kunwar 132		Pb	Powder used as tonic.	This study	Medicinal
*Epipactis helleborine* (L.) Crantz Kunwar 133		Ra	Juice consumed to cure insanity and gout.	[11]	Medicinal
*Eria spicata* (D.Don) Hand.-Mazz. Subedi 403	Parajivi	Pb	Powder consumed during stomach ache, paste applied externally against headaches.	[11]	Medicinal
*Eulophia dabia* (D.Don) Hochr. Kunwar 134	Hatti paila	Rz	Powder consumed against coughs and heart trouble, also used as tonic and appetizer.	[1,11]	Medicinal
*Eulophia spectabilis* (Dennst.) Suresh (syn. *Eulophia nuda* Lindl.) Kunwar 135	Amarkand	Tb	Powder used against worm infestation, scrofula, blood disorders, bronchitis and as appetizer.	[11]	Medicinal
*Flickingeria fugax* (Rchb.f.) Seidenf. Kunwar 140	Jiwanti	Ep	Powder used as tonic against general debility and as stimulant.	This study	Medicinal
*Flickingeria macraei* (Lindl.) Seidenf. Subedi 319	Jiwanti	Ep	Paste used against snake bites, general debility, as stimulant and demulcent.	[1,25]	Medicinal
*Gastrodia elata* Blume Subedi 421		Tb	Dried powder used as tonic and for treating headaches.	[11]	Medicinal
*Gymnadenia orchidis* Lindl. Kunwar 141		Tb	Powder used to treat gastric, urine and liver disorders.	[11]	Medicinal
*Habenaria intermedia* D.Don Subedi 422	Riddhi	Ra, Lf	Powder used for blood diseases.	[3]	Medicinal
*Habenaria pectinata* D.Don Kunwar 141	Seto musli	Tb, Lf	Leaf juice applied on snake bites. Tuber used against arthritis.	[3]	Medicinal
*Luisia trichorrhiza* (Hook.) Blume Subedi 320	Arjona	Lf	Paste applied externally to treat muscular pain.	[11]	Medicinal
*Luisia tristis* (G. Forst.) Hook.f. (syn *Luisia zeylanica* Lindl.) Subedi 423	Bori jhaar	Ep	Juice used for treating chronic wounds.	[11]	Medicinal
*Malaxis muscifera* (Lindl.) Kuntze Kunwar 142	Jivaka	Pb	Paste applied during diathesis, burning sensation, fever, on sores and as tonic.	This study	Medicinal
*Otochilus albus* Lindl. Subedi 370	Aankhle laharo	Ep	Powder used as tonic.	This study	Medicinal
*Otochilus lancilabius* Seidenf. Kunwar 107	Aankhle laharo	Ep	Paste applied to fractured and dislocated bones.	[6]	Medicinal
*Papilionanthe teres* (Roxb.) Schltr. Subedi 424	Harjor, Thurjo	Pb, Lf	Paste externally applied to treat dislocated bones.	[7]	Medicinal
*Pholidota articulata* Lindl. Subedi 368	Hadjor	Ep	Paste applied on fractured bones and consumed as tonic.	This study	Floricultural, medicinal
*Pholidota imbricata* Lindl. Subedi 367	Thurjo, Patharkera	Pb	Paste consumed to relieve fever and powder as tonic.	This study	Medicinal
*Pholidota pallida* Lindl. Subedi 369	Thurjo, Patharkera	Rh, Pb	Paste used to relieve fever, powder to induce sleep and to cure abdominal pain, juice used for navel pain.	This study	Floricultural, medicinal
*Platanthera edgeworthii* (Hook.f. ex Collett) R.K.Gupta (syn. *Habenaria edgeworthii* Hook.f. ex Collett) Kunwar 145	Riddhi	Rh, Lf	Powder or paste consumed to cure blood diseases and for cooling.	[3]	Medicinal
*Pleione humilis* (Sm.) D.Don Kunwar 108	Shaktigumba	Pb	Paste applied on cuts and wounds. Powder used as tonic.	[7,21]	Floricultural, medicinal
*Pleione praecox* (Sm.) D.Don Kunwar 109	Shaktigumba	Pb	Dried powder consumed with milk as tonic and energizer. Paste externally applied on cuts and wounds.	[22], This study	Floricultural, medicinal
*Rhynchostylis retusa* (L.) Blume	Chadephuul, Dhogegava	Ep	Juice of roots applied to cuts and wounds. Leaf powder used to cure rheumatic diseases. Dried flowers as insect repellent and to induce vomiting.	[1,7,10,22]	Floricultural, medicinal
*Satyrium nepalense* D.Don	Mishri, Thamni	Tb	Dried tubers consumed as tonic against dysentery. Juice consumed against fever and applied on cuts and wounds.	[7,21]	Medicinal
*Spiranthes sinensis* (Pers.) Ames Subedi 451		Tb	Powder consumed against headaches as tonic and energizer.	[19,26]	Medicinal
*Thunia alba* (Lindl.) Rchb.f.	Golaino	Ep	Paste used on fractured bones.	[25]	Medicinal
*Vanda cristata* Wall. ex Lindl. Subedi 201	Vhagute Phul, Thuur	Ra, Lf	Root paste applied to boils and to treat dislocated bones. Leaf powder used as expectorant, paste applied to cuts and wounds.	[7,21]	Floricultural, medicinal
*Vanda tessellata* (Roxb.f.) Hook.ex G.Don (syn. *Vanda roxburghii* R.Br.) Subedi 467	Parajivi, Rasna	Ra, Lf	Root used as antidote for scorpion stings, and remedy of bronchitis and rheumatism. Paste of leavesused to treat fevers.	[3]	Floricultural, medicinal
*Zeuxine strateumatica* (L.) Schltr. Subedi 200	Kansjhar	Rt	Dry powder used as tonic.	This study	Medicinal

^a^Vouchers are deposited at TUCH; ^b^Fl, flowers; Lf, leaves; Pb, pseudobulb; Ra, roots; Rz, rhizome; Tb, tubers; Ep, entire plant.

### DNA barcoding of illegally traded orchids

DNA barcoding is a powerful tool used to control trade in species placed on either CITES Appendix I or II [27-29] and to trace cross-border wildlife crime [30].

Purchased orchid samples that were unidentifiable by morphology, and failed to flower in the experimental garden were selected for DNA barcoding identification at the Laboratory of Plant Systematics, Central Department of Botany, Tribhuvan University, Kathmandu. The following methods and materials were applied: Material was ground to powder in a mortar with liquid nitrogen. Total genomic DNA was extracted from 40-100 mg of powder using the DNeasy Plant mini kit (Qiagen Inc.). Parts of the plastid *matK* gene and nuclear nrITS regions were amplified using the primers-19 F; 881R, 731; 2R, and 101 F; 102R, respectively [31]. Polymerase chain reactions were carried out on a PXE 0.2 Thermocycler (Applied Biosystems) in a 25 μl volume containing 0.1-50 ng of genomic DNA, 0.1 M of each primer, 10 M of each dNTP, Qiagen PCR buffer (50 mM KCl, 10 mM Tris–HCl pH 8.7, 1.5 mM MgCl_2_) and 1.5 units of Taq DNA polymerase (Qiagen, Inc.). The thermal cycling profile started with a 5 min denaturation step of 94°C, then comprised 35 cycles each with 20 s denaturation at 94°C, 20 sec annealing at 48-51°C and 45 s elongation at 72°C, and the program ended with 5 min extension at 72°C. Amplification products were separated on a 1% agarose/TAE gel, purified using the QIAquick PCR Purification Kit (Qiagen Inc.) and sequenced on an ABI 3730xl automated sequencer by Macrogen (South Korea) using standard dye-terminator chemistry following the manufacturers protocols (Applied Biosystems). Complementary strands were assembled and edited with Sequencer version 4.01 (Gene Codes Corporation).

NCBI GenBank BLAST searches [32] were used to match DNA sequences generated in this study with those already deposited in the database. Species names were assigned only in cases of a sequence similarity of 100%. DNA sequences generated were submitted to GenBank (accessions JF422074 - JF422082; Table 1).

## Results and discussion

### Medicinal orchids of Nepal

Sixty species were reported to be used for 38 different ailments (Table 1), representing 15% of the total number of orchids described from Nepal. A recent literature review by Acharya and Rokaya [9] found 82 medicinal orchid species reported from Nepal, 47 of those were also found in this study focusing on a limited area. Hossain [5] in a global literature review on medicinal orchids shows that a total of 129 species are being used for different therapeutic purposes. Eighty-two medicinally used orchids in Nepal imply that the diversity of traditional orchid species in the country is exceptionally high. The high number could be explained by the fact that our study is the first ethnobotanical survey focusing solely on orchids. In addition, the use of DNA barcoding enabled more accurate species identification of sterile material than can be achieved in morphological studies (Table 2).

**Table 2:** DNA barcoding of sterile medicinal orchids

**Medicinal orchid**	**DNA barcoding**			
Scientific name Voucher number	Marker	Voucher sequence	Reference sequence	Citation for reference sequence
*Coelogyne cristata* Lindl. Subedi 224	nrITS			[31]
	*mat*K	JF422077	AF302707	
*Coelogyne fimbriata* Lindl. Subedi 225	nrITS	JF422074	AF302745	[31]
	*mat*K	JF422078	AF302710	
*Coelogyne stricta* (D. Don) Schltr.; Subedi 314	nrITS	JF422075	AF302757	[31]
	*mat*K	JF422079	AF302722	
*Pleione praecox* D. Don Kunwar 109	nrITS	JF422076	AF461491	[33]
	*mat*K	JF422082	AF503742	

Acharya and Rokaya [9] recorded 82 medicinal orchid species for Nepal, 34 of which were not recorded in this study, whereas this study recorded 12 additional species. The combined total of both studies comes to 94 medicinally used orchid species. The majority of these are epiphytes, a fourth is terrestrial, and just a few are lithophytes. *Coelogyne*, *Dendrobium*, *Cymbidium*, *Bulbophyllum*, *Habenaria*, *Malaxis* and *Pholidota* are the genera of which most species are being used as traditional medicines. Other reported uses of these medicinal orchids are fodder (25), vegetables (6) and ritual and ceremonial uses (6) [10].

The most common vernacular names for orchids are *Sungava* and *Sunakhari*. In addition 23 vernacular names for orchids were recorded to be used by local communities in different parts of Nepal (Table 1). Among these most common are: *Thuur* or *Thurjo* (moss-like plants growing on tree trunks), *Parajivi* (parasitic plant), *Bankera* (pseu-dobulbs shaped like a wild banana), *Banaduwa* (ginger-like), *Chandigava* (silver-coloured flowers), *Shaktigumba* (pseudobulbs providing energy) and *Chadephul* (flowers inducing vomiting). The vernacular names reflect the vast knowledge of local communities with regard to orchid growing habits, habitats and their potential uses.

Major reported local uses include aphrodisiacs, energizers, and treatments of skin burns, fractured or dislocated bones, headaches, fever, and wounds. Other uses include insect repellent, blood purifier, skin fungi, snake and scorpion bite antidote, inducement of abortions, and recovery from childbirth. Orchids are mainly used as paste, powder or juice, solely or mixed with milk, honey or wheat flour. Orchid extracts are either consumed orally or applied externally. A widespread local use of *Coelogyne* is to eat freshly cut slices of the pseudobulb as a thirst quencher.

### Wild orchid species in trade

From the total of 60 species of wild orchids recorded as traded from the study sites, 28 species were exported both for medicinal and floricultural purposes, and 32 species for medicinal purposes only. Multiple use-values exacerbate the threat of overexploitation for these species. For medicinal purposes, species belonging to the genera *Acampe*, *Aerides*, *Coelogyne*, *Crepidium*, *Dactylorhiza*, *Dendrobium*, *Gastrodia*, *Eulophia*, *Flickingeria*, *Otochilus*, *Pholidota*, *Satyrium* and *Vanda* are most heavily exploited based on the number of times these were cited by the respondents. *Acampe praemorsa*, *Aerides multiflora*, *Bulbophyllum careyanum*, *Coelogyne cristata*, *Co*. *nitida*, *Crepidium acuminatum*, *Dactylorhiza hatagirea*, *Dendrobium aphyllum*, *De*. *crepidatum*, *De*. *eriiflorum*, *De*. *moschatum*, *Eulophia spectabilis*, *Flickingeria fugax*, *Gastrodia elata*, *Otochilus albus*, *Pholidota pallida*, *Ph*. *imbricata* and *Vanda cristata* are the most wanted species for rituals. *Coelogyne cristata*, *Co*. *flaccida*, *Co*. *nitida*, *Cymbidium iridioides*, *Dendrobium densiflorum* and *Vanda cristata* are most widely exploited as cut flowers.

### Orchid collectors and collecting practices

Collecting wild orchids was predominantly done by local youths, women and children, and a total of 42 collectors were recorded across the study sites. At Dakshinkali, at least 18 local collectors were involved in orchid collection, supplying the 10 local vendors with orchids. The vendors themselves were sometimes also involved in collecting wild orchids. Some local collectors reported to have been involved in orchid collection and selling for more than 25 years.

Medicinal orchids were usually harvested from December up to April with a peak period from January to March. For floriculture, the collection period was found to be throughout the year depending on the availability of flowering individuals. Collectors reported to search far and wide for orchids, frequently traveling more than 10 km on foot through the forest. Epiphytic orchids growing high up in tree canopies were collected by felling the trees if feasible, and preferentially collected in clumps. Terrestrial orchids were collected by unearthing the tubers to take the entire plant.

**Figure 2:**
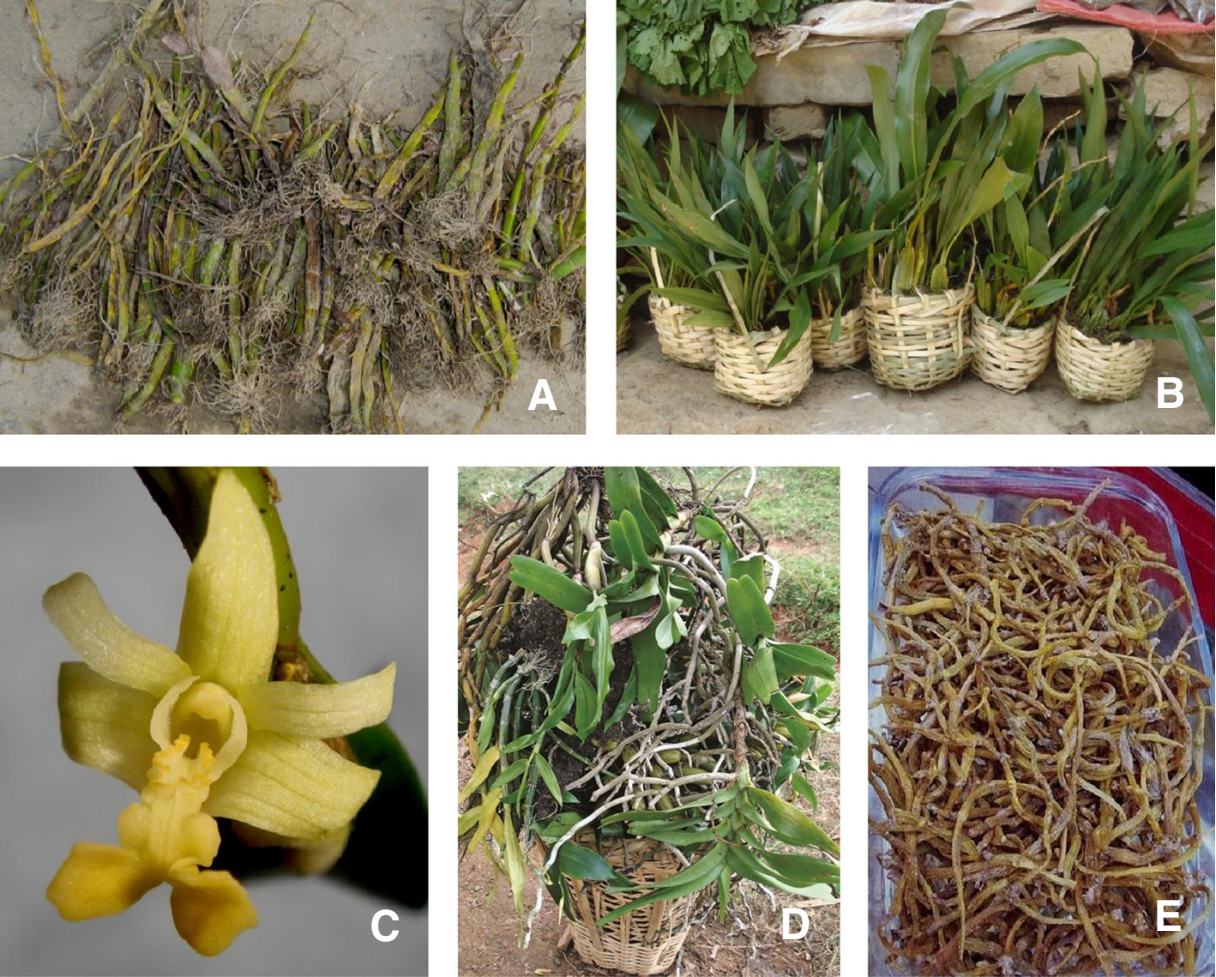
**Trade in illegally collected medicinal orchids in Nepal.** 2**A**. *Dendrobium eriiflorum* (photograph: B. Kunwar). 2**B**. *Coelogyne* species in market outlets (photograph: A. Subedi). 2**C**. *Flickingeria fugax* (photograph: A. Subedi). 2**D**. *Aerides* and *Dendrobium* species in traditional bamboo baskets (photograph: B.B. Raskoti). 2**E**. *Dendrobium eriiflorum* originating from Nepal in Hong Kong supermarkets (photograph: Anonymous).

Collection of wild orchids usually started once a purchase order was received from middlemen. These persons usually stayed nearby orchid collection sites throughout the collection period. Sometimes, the collectors received advance payments. The middlemen usually came from distant districts or even abroad, and provided printed photographs of desired species or small samples of life orchids, and asked collectors to collect similar-looking plants. An example of such a photograph was retrieved from a middleman who received it from international traders based in Hong Kong (Figure 2E). Local people collected all orchids found, also when these did not resemble the species on the photographs provided by the middlemen. None of the orchids collected were discarded at the selling points. Most collectors spent an average of 5-6 h per day in the forest. They carried the orchids in bamboo baskets (Figrue 2A-D) or in jute sacks to the nearest selling points. Over the past 15 years, large-scale orchid collection in Nepal has increased year-on-year based on the traded volumes cited by the respondents.

### Wild orchids market outlets

Dakshinkali, 22 km from Kathmandu, is the center of wild orchid trade in Nepal, and orchids have been sold for over 25 years. Dakshinkali has at least 10 vendors that are specialized in wild orchid trade. Another significant trade hub is Godavari, near Kathmandu, but orchid trade here has gradually declined over the last five years. Dakshinkali is famous for its historic Hindu Kali temple, and every year up to 400.000 pilgrims visit this temple, and purchase wild orchids, which play an important role in ceremonial rituals. Many hotel owners in Kathmandu buy wild orchids at Dakshinkali. These orchids can easily be recognized by their traditional woven bamboo baskets that are specially made for the purpose of selling wild orchids, and not found elsewhere in Nepal.

The east–west highway of the tropical part of central Nepal is another very active site for orchid trade. No fixed orchid selling locations are present here, but every year, the middlemen and/or local traders inform the collectors where the orchids should be brought. At these transitory trade points, the orchids are weighed and traded, with large volumes loaded onto trucks or tractors and transported illegally to India or China.

### Wild orchids trade volume and local income

The peak season for orchid trade at Dakshinkali is from July to October. In this period in the year 2008-2009, each live orchid vendor sold an average of 15-20 pots per day, which averages to 2-2.5 kg of orchids. Extrapolated to yearly trade per vendor this averages to 4.4 tons of orchids per year (2.25 kg × 17.5 pots × 7 days × 16 weeks). The vendors sold both vegetative and flowering orchids, but the latter fetched the highest prices. Popular species such as *Dendrobium densiflorum*, *Coelogyne cristata*, *Cymbidium iridoides* and *Cymbidium erythraeum* traded at the highest values. The price of orchids for floricultural purposes was highly variable and fluctuating, but averaged USD 1.0-1.5 per pot. This average price allows us to make a rough estimate of the annual income per vendor from orchid trade: 17.5 pots x 1.25 USD × 7 days × 16 weeks = 2450 USD.

Local medicinal orchid traders and middlemen reported that orchid trade had declined recently due to the arrest of a number of illegal traders. Collectors were reported to earn an average of USD 2 per kg for medicinal orchids with prices varying between USD 1.5-2.5 depending on the species and quality of the orchids. Based on the interviews we estimate an annual trade of 5 tons for 2008-2009, yielding a combined annual total for Dakshinkali of 9.4 tons of wild orchids for that year.

Detailed export prices of wild orchids collected at the study sites could not be assessed since the traders refused to provide these data. One trader informed us that processed *Dendrobium eriiflorum* sold for 10,000 Hong Kong dollars (~ 1300 USD) per kg. This is in line with the general conception that wild orchids from Nepal fetch higher prices internationally than on the domestic market.

### Legal and illegal trade destinations of Nepalese orchids

Interviews with collectors, middlemen and local traders revealed that most of the wild orchids collected in Nepal are exported to India and China, and occasionally to Hong Kong. None of the actors involved had received permission from local authorities. The local traders mostly exported raw or occasionally semi-processed, dried and cleaned, products. Our findings support previous reports about illegal trade in Nepalese orchids [13,15]. Shakya et al. [14] reported that wild orchids from Nepal were exported to European countries for floricultural purposes, with none of the exported species grown at nurseries. Nepalese newspapers frequently report cases in which orchid smugglers are arrested with huge quantities of wild orchids for export to China.

## Discussion and conclusions

### Sustainable use of medicinal orchids

Collection and use of wild orchids of Nepal is deeply engrained in the traditional livelihoods of local communities. They form an important part of the traditional health care system and provide a substantial income to subsistence farmers. An increasing number of species are now illegally traded in bulk volumes to some of the most rapidly growing economies in the region, China, India and Hong Kong. This illegal trade creates a severe threat to wild orchid populations in Nepal [34], urging development of alternative strategies for sustainable exploitation. We advocate development of sustainable orchid enterprises focusing on medicinal orchid species grown from cuttings and seed. Cultivated of orchids for raw ingredients of herbal medicine is a niche in the international orchid market that is still relatively undeveloped, and deserves further exploration [2,35,36].

Artificial propagation of orchids has the potential to reduce illegal collecting in the wild through wider availability of stock material, and can also provide large numbers of plants within a short period of time. Artificially propagated plants often have the advantage of being more vigorous than wild collected stock, have a higher survival rate and contain higher contents of compounds with pharmacological effects [37,38]. The establishment of a sustainable national orchid industry based on low cost *in vitro* propagation could be beneficial to the conservation of endangered orchids, and for several species of wild-collected Nepali orchids these techniques have already been developed [39-41]. The potential disadvantage of undercutting local collectors and traders is that as their livelihoods are jeopardized, they are forced to diverge into other sources of supplementary income, such as the collection of other medicinal plant species.

### Policies for protection of wild orchids in Nepal

All wild orchids of Nepal are protected under Appendix II of the Convention on International Trade in Endangered species of Wild Fauna and Flora (CITES). The Forest Act 1993, and Forest Regulations 1995, and amendment in 2001 specified all orchids in Nepal as protected. However, contradicting its own policies, the Government of Nepal published a notification on April 14th, 2008 permitting collection of wild orchids for trade. The absence of clear guidelines on sustainable harvesting and weak enforcement of policies could explain the recent increase in illegal trade in orchids [22].

### DNA barcoding for identification of sterile orchids

Sterile plant parts sold at local markets can be identified to species level using DNA barcoding. DNA barcoding is increasingly applied for plant species identification [42,43]. This method can both provide the taxonomic identity of samples analyzed, and - if the markers employed are sensitive enough - elucidate the geographical origin of the collected species [28]. For the former scenario, DNA barcoding is ever-increasingly facilitating monitoring trade of CITES-listed species [27]. For the latter, the method is more and more used to trace and substantiate cross-border wildlife crimes [30,44]. We recommend a wider application of DNA barcoding for identification of orchid species in illegal export, as it enables identification of material that is unidentifiable by morphology alone.

## Endnote

^a^Footnote: author names are provided for all species in Table 1. Species not included in Table 1 have author names included in the manuscript body.

## Competing interests

The author(s) declare that they have no competing interests.

## Authors’ contributions

AS, BG, and RC conceived the research. AS and BK were responsible for field research and interviews. AS, BK, BG and RC identified the herbarium vouchers; AS, YC, YD, TA and HB processed the data and performed the quantitative analysis. AS, HB and BG contributed to the manuscript. All authors have read and approved the final manuscript.
